# Reply to: “Pitfalls in identifying active catalyst species”

**DOI:** 10.1038/s41467-020-18193-2

**Published:** 2020-09-11

**Authors:** Xavier Isidro Pereira-Hernández, Andrew DeLaRiva, Valery Muravev, Deepak Kunwar, Haifeng Xiong, Berlin Sudduth, Mark Engelhard, Libor Kovarik, Emiel J. M. Hensen, Yong Wang, Abhaya K. Datye

**Affiliations:** 1 Voiland School of Chemical Engineering and Bioengineering, Washington State University, Pullman, WA 99164 USA; 2 Department of Chemical and Biological Engineering and Center for Micro-Engineered Materials, University of New Mexico, Albuquerque, NM 87131 USA; 3 Laboratory of Inorganic Materials and Catalysis, Schuit Institute of Catalysis, Eindhoven University of Technology, P.O. Box 513, 5600 MB Eindhoven, The Netherlands; 4 Environmental Molecular Sciences Laboratory, Pacific Northwest National Laboratory, Richland, WA 99354 USA; 5 Institute for Integrated Catalysis, Pacific Northwest National Laboratory, Richland, WA 99354 USA

**Keywords:** Catalytic mechanisms, Heterogeneous catalysis, Chemical engineering

**Replying to** J. Ren & Y. Chen *Nature Communications* 10.1038/s41467-020-18192-3 (2020)

In Pereira-Hernández et al.^1^, we reported the influence of the high-temperature vapor-phase synthesis method (also called atom trapping, or AT) on the activity for CO oxidation of a Pt/CeO_2_ catalyst, compared to a conventional synthesis method (strong electrostatic adsorption, or SEA). The findings suggest that the AT method leads to increased activity compared to the SEA method, and this is related to improved redox properties of the support at low temperature. Recently, Ren and Chen^2^ questioned the interpretation of the results and suggested alternative explanations for the findings. However, as addressed in this paper, we are firmly of the opinion that the original analysis, results, and conclusions provided in Pereira-Hernández et al.^1^ are valid and accurately explain the phenomena observed.

The AT catalyst is significantly more active than the SEA catalyst at 50 °C (Fig. 1 in Pereira-Hernández et al.^1^). Ren and Chen^2^ suggested that the difference in activity between the two catalysts might be related to a difference in metal dispersion. However, Supplementary Fig. 4^1^ shows that the mean particle sizes for the AT and SEA catalysts are 1.68 ± 0.3 and 1.58 ± 0.33 nm, respectively, confirming that the two catalysts in the original paper have a similar Pt dispersion. A similar particle size would also imply a similar interface area. Hence, the difference in reactivity arises from the nature of the ceria (different ceria redox properties at 50 °C, confirmed by NAP–XPS). The major difference between AT and SEA is the activation of the ceria support. In our recent publication^3^, we further demonstrated that CO adsorbed on Pt in the AT sample reacts quickly at 70 °C, if oxygen is available in interfacial sites. On the other hand, if the interfacial oxygen is depleted, CO is bound strongly. This proves unequivocally that the interfacial sites in the AT sample are necessary for low-temperature CO oxidation.

**Fig. 1:**
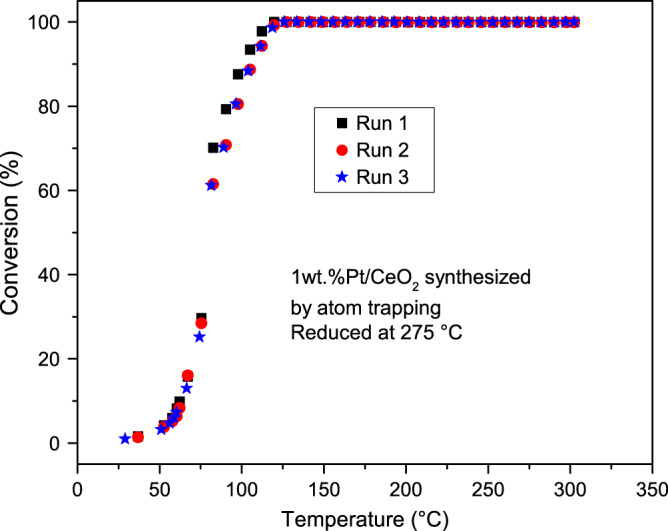
Sequential CO oxidation. Sequential CO oxidation light-off curves up to 300 °C using a 1 wt%Pt/CeO_2_ catalyst synthesized by atom trapping and further reduced at 275 °C with CO, as specified in Pereira-Hernández et al.^1^.

Moreover, Ren and Chen^2^ express a concern that the QMS results during the NAP–XPS experiments do not show a significant signal for CO_2_ at temperatures where the packed-bed reactor and CO-TPR show significant CO_2_ production. The low signal can be easily explained since the flow geometry in the NAP–XPS experiments is not optimized for accurate kinetic measurements. Gases flow over the catalyst bed, and the residence time is much lower than the CO-TPR experiment. The CO-TPR is performed at ambient pressure versus 2 mbar in NAP–XPS. The residence time in the NAP–XPS experiments is about two orders of magnitude less than that in the CO-TPR experiments, leading to about two orders of magnitude lower CO_2_ concentration, which makes CO_2_ more difficult to detect. In addition, CO flows over the catalyst bed during NAP–XPS measurements, likely resulting in external mass transfer limitation, making CO_2_ detection even more difficult. In other words, the geometry of the NAP–XPS cell (gas flows over the sample) and location of the QMS (differential pumping of the lens system, not the outlet of the cell) are not designed for reactivity measurement. QMS data from NAP to XPS are used to verify the composition of the reactant gases fed to the catalyst in the cell.

Ren and Chen^2^ comment on the differences between the pretreatments used for CO-TPR versus NAP–XPS, and suggest that the oxidative treatment converts the Pt to the oxide that gets reduced to form CO_2_. If so, we would have expected similar reduction behavior for the AT and SEA catalysts during CO-TPR since the amount of Pt in both catalysts is the same. The fact that CO_2_ is formed at lower temperatures on the AT catalyst is a result of the enhanced reactivity of the ceria support in the AT catalyst. The catalyst pretreatment for CO-TPR versus NAP–XPS cannot account for this difference.

Furthermore, the geometry of the NAP–XPS cell (gas flows over the sample) and location of the QMS (differential pumping of the lens system, not the outlet of the cell) are not designed for activity measurement. No reactivity data can be extracted with this type of system. Its function is only to provide qualitative trends. CO conversion was also low at 50 °C (<30%) even in a flow reactor without external mass transfer limitation (Fig. 1 in Pereira-Hernández et al.^1^). Therefore, there is no contradiction in the inability to detect CO_2_ during the NAP–XPS experiments.

In addition, Ren and Chen^2^ express concerns about the NAP–XPS quantification of different Ce species due to our use of a Shirley background. Quantification of the Ce^3+^/Ce^4+^ ratio from fitting of the Ce3d line is challenging and requires experience and use of advanced models. However, spectra fitting with the model, which takes an account of the asymmetry of the u and v components and Shirley-type background, is justified as shown by recognized surface science groups specializing on ceria model systems^4–9^. While the choice of the fitting model and the background do matter, consistency of fitting throughout the experiments/samples (using the same measurement parameters) limits the uncertainty only to the absolute Ce^3+^ concentration, with no effect on the overall speciation on which the conclusions are based^5^.

The quote used by Ren and Chen^2^ “*However, the 2009 report*^10^ *cautioned about Shirley background and further stated that decomposing the complicated spectrum is “partly ambiguous in principle*”” leaves out the following statement from Skala’s seminal work. Immediately after the phrase quoted above, Skala et al. conclude: “*Despite these remarks we obtained a high-quality and consistent fit—better than many of those already published in the literature—and quite simple at the same time*”. We therefore feel justified in our use of the Shirley background for fitting the data.

In addition, in an extensive overview recently published by Paparazzo^11^, the fitting provided by Skala (and on which our fitting is based) was assessed as “both accurate and consistent”.

Ren and Chen^2^ suggest that the different Pt/Ce ratios between the two catalysts indicate different dispersion. However, it is important to take into account that the AT catalyst was pretreated at 800 °C in air for 10 h, leading to decreased surface area. This explains a higher Pt/Ce ratio of ~0.030 on the AT catalyst versus ~0.015 on the SEA catalyst, despite similar particle sizes of Pt. The presence of atomically dispersed Pt^2+^ on the AT catalyst further increases the Pt/Ce ratio.

Ren and Chen^2^ raise additional concerns about the activity/stability of the AT catalyst based on the QMS data during the NAP–XPS experiments, suggesting that the activity is lost in about 30 min. To address this concern, we point to Supplementary Fig. 2 of our recent publication^1^, which illustrates the repeatability and stability of the AT catalyst. Five consecutive runs up to ~140 °C were performed, and no loss of activity was observed. We recently repeated these measurements using CO reduction, and extended the reaction temperature to 300 °C. No deactivation was observed, as shown in Fig. 1.

Ren and Chen^2^ point out that there might be a contradiction in the Pt(0) content evolution throughout the NAP–XPS experiments; however, under switches from CO + O_2_ to CO, not only the coverage of CO on Pt would be different, contributing to the significantly different spectrum, but also chemical potential of the system substantially changes, leading to reconstruction of the surface of Pt NPs^12^. That is why we not only observe a reversible minor but significant shift in BE upon switches, but also a difference in intensity of the component. So, the picture inferred from Pt(0) can be quite complicated and can be the subject of further investigations. This is why we clearly stated in the paper^1^ “Further exposure to CO and CO + O_2_ environments at 50 °C does not change significantly the fraction of Pt^0^ species in the catalyst” for Figs. 5 and 6.

The results shown in Figs. 5 and 6^1^ were performed in a SPECS NAP–XPS system, while the results in Supplementary Fig. 10^1^ were performed in a Kratos AXIS Ultra spectrometer. The latter allows treatment of the catalyst at atmospheric pressure, but has the potential issues caused by sample transfer, while the pressure was limited to 10 mbar in the NAP–XPS system. Therefore, comparisons of two catalysts should be performed using the same equipment. In both cases, however, the AT catalyst shows more Ce^3+^ species after reduction than the SEA catalyst, implying a higher reducibility of ceria, which is consistent with the explanation of an improved supply of oxygen species to the Pt surface.

As discussed above, the choice of the exact model (linear background or Shirley type) might alter the absolute values obtained from the fit. However, if the same processing approach is used throughout all spectra, the trend will remain valid. The comprehensive overview of fitting models for Ce3d core line made by Paparazzo^11^ concluded that the fitting provided by Skala (and on which our fitting is based) was assessed as “both accurate and consistent”, further justifying our approach.

Ren and Chen^2^ suggest that the SEA catalyst might be more active due to the higher amount of gas- phase CO_2_ seen during the DRIFTS measurements. However, catalyst reactivity cannot be inferred from IR spectra of the products, especially in the CO_2_ region because of interference from atmospheric CO_2_ outside the DRIFTS cell. For example, the region for CO_2_ gas phase in Fig. 3d^1^ does not change during CO oxidation, He desorption, and O_2_ flow. If this signal was related to CO_2_, the peak should have disappeared once the CO was stopped, which was not the case. To clarify this, QMS results for the AT and SEA catalysts during DRIFTS experiments (which were not included in the original manuscript) are shown in Fig. 2. Both catalysts exhibit low activity for CO oxidation at 50 °C, which can be explained by the flow dynamics in the DRIFTS cell. However, increasing temperature to 125 °C leads to clearly different activities between the AT and SEA catalysts.

**Fig. 2:**
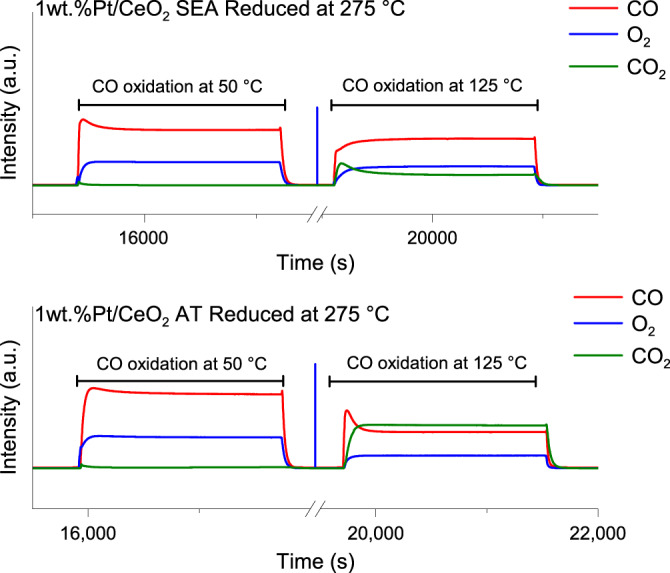
QMS signals for CO, O_2_, and CO_2_ during CO oxidation reaction that was performed and monitored by DRIFTS. Top: 1 wt.%Pt/CeO_2_ catalyst synthesized by SEA. Bottom: 1 wt.%Pt/CeO_2_ catalyst synthesized by AT. Both catalysts were reduced at 275 °C with CO, as specified in Pereira-Hernández et al.^1^.

Ren and Chen^2^ proposed calculating TOF using “active ceria sites”. It is understandable and logical that calculation of TOF requires counting the number of active sites. While the sites at the interface play a critical role in this reaction, quantifying the number of these sites is very difficult. Simply using the perimeter of a nanoparticle is not accurate due to variations in particle shape compounded by the difficulty in accurate determination of size and interfacial area in subnanometer particles. Previous work by Cargnello et al.^13^, which focused on investigating the role of particle size (and interface sites), used the total amount of Pt for normalizing reactivity. This is also the approach used by all studies on single-atom catalysts (SACs); hence, we calculate TOF based on the total number of Pt atoms.

## Data Availability

The authors declare that all the data supporting the findings of this study are available within the paper or from the corresponding author(s) upon request.
